# The role of human papillomavirus type 16 and the fragile histidine triad gene in the outcome of cervical neoplastic lesions

**DOI:** 10.1038/sj.bjc.6602253

**Published:** 2004-11-30

**Authors:** G Terry, L Ho, P Londesborough, P Cross, A Lopes, J Monaghan, J Cuzick

**Affiliations:** 1 Department of Epidemiology, Mathematics and Statistics, CRUK, Wolfson Institute, Queen Mary & Westfield University, Charterhouse Square, London EC1M 6BQ, UK; 2 Department of Cyto-Histopathology, Gateshead Health NHS Trust, Queen Elizabeth Hospital, Sheriff Hill Gateshead NE9 6SX, UK; 3 Northern Gynaecological Oncology Centre, Gateshead Health NHS Trust, Queen Elizabeth Hospital, Sheriff Hill Gateshead NE9 6SX, UK

**Keywords:** HPV16, FHIT, cervical cancer, CIN1

## Abstract

The presence of high-risk human papillomavirus, loss of heterozygosity on chromosome 3p and fragile histidine triad gene expression were assessed as potential markers of cancer and CIN progression in 83 cervical cancers and 74 cervical intraepithelial neoplasia grade 1 lesions. Human papillomavirus type 16 was an indicator of vascular involvement in cancers. Loss of heterozygosity, especially in the fragile histidine triad gene intron 5, was an indicator of high-grade tumours, greater tumour depth and lymph node involvement. Abnormal fragile histidine triad gene expression was more frequent in cervical intraepithelial neoplasia grade 1 lesions with increased risk of disease progression.

Cervical cancer is the second most common cancer in women worldwide and is nearly always associated with one or more of the human papillomavirus types which have been shown to carry a high oncogenic risk (HR HPV) (Munoz *et al*, 2003). Most HR HPV infections are transient and resolve spontaneously. High-grade lesions (cancers and cervical intraepithelial neoplasia grades 2 and 3 (CIN2/3)) are more likely to result from persistent infections, particularly with HPV16 and/or high viral load (Ylitalo *et al*, 2000). HR HPV infection represents only one of many events occurring early in the development of cervical malignancy, however, and multiple subsequent genomic and epigenomic changes are believed to be required.

Loss of heterozygosity (LOH) is an early indicator of genomic instability, which increases the risk of malignancy. In many human cancers, LOH is frequently observed in the short arm of chromosome 3 (3p) (Naylor *et al*, 1987; Hibi *et al*, 1992; Druck *et al*, 1995; Kastury *et al*, 1996; Li *et al*, 1997). For cervical neoplasia, the chromosomal region of particular interest is 3p14.2 because it includes two known HPV16 integration sites (Thorland *et al*, 2000; Corbin *et al*, 2002) as well as the most commonly disrupted genomic fragile site, FRA3B (Glover and Stein, 1988). This site is sensitive to environmental carcinogens (Schantz *et al*, 2000) and is located between intron 3 and intron 7 of a putative tumour suppressor gene, fragile histidine triad (FHIT), which has proapoptotic activity (Ji *et al*, 1999; Sard *et al*, 1999; Dumon *et al*, 2001; Roz *et al*, 2002). One HPV insertion site is located in intron 4 and the other is centromeric to the FHIT gene. The close proximity of these genetic elements permits genomic events arising from breaks and improper repair of such breaks in this region to be readily monitored.

Loss of heterozygosity in 3p and reduced FHIT expression have been reported in cervical cancer and precancer lesions (Wistuba *et al*, 1997; Birrer *et al*, 1999; Chung *et al*, 2000; Connolly *et al*, 2000; Guo *et al*, 2001; Acevedo *et al*, 2002) but, because of small sample numbers or because of differences in the 3p loci selected for LOH analysis, it is not clear if any regions of 3p are consistently deleted or if reduction in FHIT protein level is dependent on these deletions. In most studies, the HR HPV genotyping was also incomplete. More importantly, the disease outcome associated with these genomic changes has not been fully investigated. An understanding of the biological significance of changes in this chromosomal region could provide additional information relevant to the outcome of cervical lesions.

In this study, we have examined 83 cervical cancers by analysis of LOH in chromosome 3p and by HPV genotyping to evaluate the association between these genetic elements and standard histopathological prognostic markers (histology type, histology grade, depth of tumour invasion, blood vessel involvement and lymph node involvement) indicative of progressive disease. Genetic markers with significant associations were then analysed in 74 low-grade CIN1 lesions to see if these markers could predict the progression of cervical disease.

## MATERIALS AND METHODS

Archival paraffin blocks of cervical biopsies and residual cervical cells after a smear preparation were obtained from 108 unselected women (mean age 50.9±16.3 years) referred to the Queen Elizabeth Hospital, Gateshead, Tyne and Wear for cervical cancer treatment. In all, 25 cancers were excluded from the study. Out of these, 11 were metastases from organs other than the cervix and 14 contained no amplifiable DNA.

A total of 78 archival paraffin blocks of loop biopsies containing only CIN1 were obtained from women (mean age 33.4±9.5 years) referred for colposcopy. Of these, 24 biopsies were from women who had been referred following a diagnostic punch biopsy showing CIN2/3. In these cases, all of the CIN2/3 lesion was presumably contained and removed in the punch biopsy since only residual CIN1 was found at all levels in the loop biopsy. These lesions are referred to as CIN1^*^ in the text. Four CIN1 biopsies contained no amplifiable DNA and were excluded from the study. Data from the remaining 83 cancers and 74 CIN1 lesions are included in this report. All histology was read by a consultant histopathologist.

### Staining for microscopy

In all, 10 serial 10 *μ*m sections were prepared from each paraffin block. Section 1 was stained with haematoxylin and eosin (H&E), section 2 was stained for proliferating cell nuclear antigen (PCNA) to confirm the proliferative activity of epithelial cells, sections 3 and 6 were stained for FHIT after epitope retrieval, sections 4 and 5 were stained with methyl-green to locate the lesion prior to micro-dissection and section 10 in CIN1 cases was scraped and used for HPV testing. For each case, the sections stained with H&E and PCNA were photographed and stored digitally. The diseased and normal tissues to be dissected were selected after agreement between two observers and dissected from the corresponding methyl-green-stained sections.

All paraffin-embedded sections were dewaxed with xylene and re-hydrated in graded alcohol and water. For the H&E stain, Gill's haematoxylin and eosin Y were used. FHIT immunostaining and assessment were carried out after epitope retrieval as described previously (Terry *et al*, 2002), except that Napthol-AS-BI (Sigma N-2125) and Fast Red TR (Sigma F-6760) were used as substrate following the supplier's instructions (Sigma) and Mayer's haematoxylin (Sigma MSH-1) was used as a counter stain. Loss or reduction in FHIT protein expression is referred to as ‘abnormal FHIT’. Proliferating cell nuclear antigen immunostaining was carried out similarly using mouse anti-PCNA antibody (clone PC10, Sigma P8825) as the first antibody, alkaline phosphatase-conjugated goat antimouse IgG antibody (Sigma A3688) as the second antibody and eosin as the counter stain. The results were assessed blind.

### Microdissection

**Figure 1:**
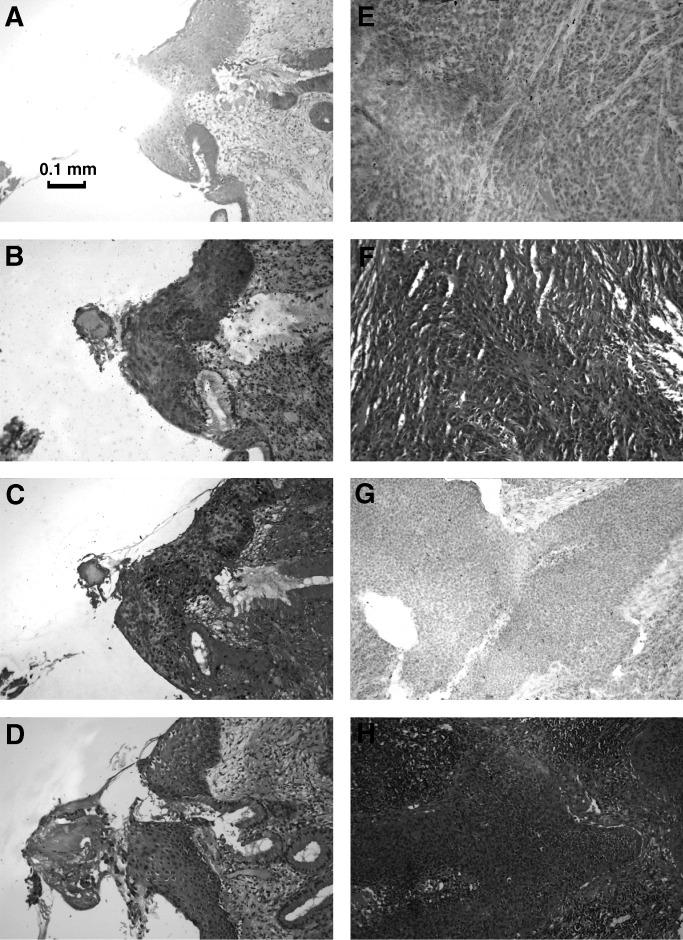
Paraffin-embedded sections of a FHIT-positive, PCNA-positive CIN1 lesion (**A**, **B**, **C**, **D**), a well-differentiated FHIT-positive cancer (**E**, **F**) and a poorly differentiated FHIT-negative cancer (**G**, **H**); stained with methyl green and microdissected (**A**), for FHIT protein (**B**, **E**, **G**), for PCNA (**C**) and with H&E (**D**, **F**, **H**).

Sterile double-distilled water was used throughout. Each section was dewaxed three times with DE-WAX (InnoGenex) for 5 min and washed with 70% ethanol. After soaking in water for 5 min, the section was stained with methyl-green (1%) in water for 1 min. Excess stain was removed by washing with water and excess water was removed by gentle shaking. While the section was still moist, normal or diseased tissue was microdissected using a 25 G needle with the help of a dissecting microscope set up inside a tissue culture hood (Figure 1A

). Special care was taken to minimise contamination with stromal tissue or lymphocytes. Dissected tissue was resuspended in 20 *μ*l T/E containing Tween 20 (1%) and proteinase K (PK, 1 mg ml^−1^). Digestion was carried out for 96 h at 55°C with three additions of fresh PK at 24 h intervals. PK was inactivated subsequently at 98°C for 10 min.

### HPV genotyping

DNA extraction from cervical cancer scrapes using PK and multiplexed type-specific PCR (mts-PCR) for 13 HR HPV types were carried out as described previously (Borysiewicz *et al*, 1996; Terry *et al*, 2002).

To determine HPV genotypes present in CIN1 biopsies, one section was scraped from the slide, digested with PK as above and amplified using the SPF primer set which can amplify a partially conserved 65 base region from at least 39 HPV types including the HR HPV types (Kleter *et al*, 1998). The amplicon was purified by electrophoresis on a low melting point agarose gel, followed by solubilisation of the agarose with agarase (AgarAce, Promega) and genotyping by direct sequencing (Cuzick *et al*, 2000) with a Cy5-labelled sequencing primer.

### Loss of heterozygosity analysis

Normal and cancer tissues microdissected from the same cancer section were compared. For CIN1 sections, epithelial cells from the PCNA-positive abnormal area adjacent to the squamo-columnar junction were compared with normal squamous epithelium. Two sections did not contain normal squamous epithelium and in these cases normal glandular epithelium was used as the control. Loss of heterozygosity at 30 3p loci was analysed in cancers using microsatellite (MS) or single-nucleotide polymorphism (SNP) markers. The chromosome locations of these markers are taken from the Human Genome Assembly version hg16 (July 2003). A combination of MS and SNP analyses allows LOH to be localised and validated.

#### Microsatellite (MS)

A total of 13 MS loci were analysed in cancers (Table 1

**Table 1:** Chromosome locations of MS and SNP loci analysed on 3p and the frequency of allelic loss (FAL) in 83 cancers

						**FAL**	
**Name**	**Type XY**	**Chromosome location (nucelotides from 3pter)**	**Region**	**Chromosome band**	**Primer pool**	**MS**	**SNP**
rs718558	GA	1469483	D3S1297, near CNTN6, contactin 6	3p26.3		2	
D3S1297		1913402	LOC391505, similar to 60S ribosomal protein L21	3p26.3	T	17	
Rs1024147	GT	2709207	D3S1297, near CNTN4, contactin 4	3p26.3			18
Rs2012053	CG	2941128	D3S1297, near CNTN4, contactin 4	3p26.2			15
D3S1304		6794241	MRPS, 36P1, mitochondria ribosomal protein S36 pseudogene	3p26.1	A	12	
D3S1286		15694136	KIAA0379 KIAA0379 protein	3p25.1	T	5	
Rs721624	GA	21625656	D3S1293, FLJ22419, hypothetical protein	3p24.3			5
D3S1293		21802013	FLJ22419, hypothetical FLJ22419 protein	3p24.3	F	2	
D3S1266		27832335	Near EOMES eomesdermin homolog (*Xenopus laevis*)	3p24.1	D	23	
Rs934328	CT	30682709	TGFBR2 TGFB receptor 2	3p24.1			0
Rs877572	CG	30701397	TGFBR2 TGFB receptor 2	3p24.1			18
D3S1211		31041792	Near LOC339896 hypothetical protein	3p24.1	T	17	
D3S1611		36929105 (MLH1 intron 12)	Near MLH1 (60k)	3p22.3	F	32	
Rs928813	GT	38489162	D3S1611, ACVR2B, RNA binding motif, s/s interacting protein	3p22.2			15
Rs1005678	CG	50169301	Near 101F6, RASSF1A, putative tumour suppressor genes, in SEMA3F, semaphorin 3F	3p21.31			32
D3S1582		54552995	CACNA2D3 Calcium channel voltage dep. a2d3 subunit	3p14.3	C	3	
Rs566884	CT	54699343	D3S1582, CACNA2D3, calcium channel voltage-dependant a2d3 subunit	3p14.3			5
Rs492234	CG	59714807 (FHIT intron 8)	FHIT Intron 8	3p14.2			13
Rs931322	GA	59883591 (FHIT intron 7)	FHIT Intron 7	3p14.2			17
Rs722070	GA	60069630 (FHIT intron 5)	FHIT Intron 5	3p14.2			26
D3S1300		60367241 (FHIT intron 5)	FHIT Intron 5; near NPCR NPC-related protein	3p14.2	C	27	
Rs2301156	GT	60515020 (FHIT intron 4)	FHIT Intron 4	3p14.2			28
Rs1562522	GA	60891272 (FHIT intron 3)	FHIT Intron 3	3p14.2			26
Rs1001788	CT	61129144 (FHIT intron 2)	FHIT Intron 2	3p14.2			0
D3S1261		68535327	Near LOC151645 prosome, macropain 26S subunit	3p14.1	D	16	
D3S1566		70132371	LOC391543 similar to KIAA1839 protein	3p13	D	8	
D3S1284		72824320	Near LOC317753 proteasome 26S non-ATPase subunit	3p13	F	23	
Rs1027833	CT	78584994	LOC51159, ROBO1, roundabout guidance receptor homologue (*Drosophila*)	3p12.3			8
D3S1595		85991642	LOC253559 nectin-like protein 3	3p12.1	A	18	
Rs1986448	AT	87086700	LOC51159, near POU1F1, POU domain, Pit1, growth hormone factor 1	3p12.1			16

) but, since the number of cells from CIN1 lesions was limited (100–500 cells), only two MS loci were tested in these cases, one in FHIT intron 5 (D3S1300) and the other 5Mb telomeric to FHIT (D3S1582). Multiplexed PCR primer pools A, C, D, F and T were used. Only pool C was used in CIN1 cases. One PCR primer for each locus was Cy5-labelled. Amplified fragments were analysed using an ALFexpress sequencer (Pharmacia Biotech) and MS peaks were quantitated using Fragment Manager software (Pharmacia Biotech). The areas under the major peaks for each MS were averaged from three independent amplifications run on different gels. Loss of heterozygosity was assigned to a cancer or CIN1 lesion, which showed an allelic ratio reduction of ⩾70% relative to the corresponding normal control.

### Single-nucleotide polymorphisms (SNPs)

In all, 17 SNP loci (Table 1) were amplified in cancer and normal DNA preparations from each cancer case and analysed by electrophoresis on 2% agarose gels to confirm successful amplification. Single-nucleotide polymorphism assays were carried out using SureScore Genotyping Kits according to the instructions of the manufacturer (Invitrogen). This test uses a two-channel ELISA-based assay to identify which of the two possible bases at the SNP position was added to a captured PCR amplicon in a single-base primer extension reaction. PCR primers and capture oligos for each SNP analysed were designed using the on-line software provided (Invitrogen). Each SNP experiment included 33 test samples, three allelic controls (supplied with the kit), three heterozygous and six homozygous human DNA controls and one control negative for human DNA all tested in triplicate. Loss of heterozygosity was assigned to a cancer sample when the absorbance ratio (405 nm/620 nm) differed from the absorbance ratio for the corresponding normal sample by more than two standard deviations.

### Statistical analysis

Association of molecular and prognostic markers was assessed using Fisher's exact test or *χ*^2^-test for trend. In the LOH analysis, the frequency of allelic loss (FAL) at a given marker was calculated as:-


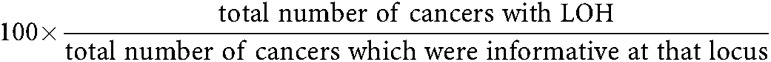


## RESULTS

### Staining and immunostaining characteristics

Figure 1 shows paraffin sections from one CIN1 lesion (a–d) and two cervical cancers (e, f, g, h), stained with: methyl-green after cancer tissue had been removed by microdissection (a), FHIT antibody (b, e, g), PCNA antibody (c) and H&E (d, f, h). Sections (b) and (e) are FHIT positive and section (g) is FHIT negative.

### Association with cancer prognostic markers

#### HR HPV

HPV16 was preferentially associated with squamous carcinoma and HPV18 with adenocarcinoma, respectively (Table 2

**Table 2:** Association of HR HPV genotypes with markers of cancer progression

				**Statistical analysis**		
**HR HPV genotype**	**Risk marker**	**Grade**	**No. of cancers positive/total**	**2*P***	**OR**	**95% CI**
16	Origin	Squamous	37/51	0.022	3.000	1.074–8.388
	Differentiation	Moderate	31/51	0.025	2.959	1.076–8.292
	Vessel involvement		32/51	0.003	4.304	1.508–12.689
Group A (16+^a^others)	Differentiation	Moderate	36/63	0.042	3.111	0.954–11.081
18	Origin	Adenocarcinoma	8/14	<0.001	10.167	2.316–44.760

Only significant associations are shown.

a Other types detected were HPV31, 33, 52 and 58, all of which are members of HPV Group A. HPV18 is classified in Group C.

). HPV16, but not HPV18, was associated with cancers showing moderate differentiation and vascular spread. No correlations between HR HPV status and FHIT protein expression were found. HR HPV genotypes detected other than HPV16 and HPV18 were HPV31, 33, 52 and 58. Like HPV16, these are all group A viruses and the group A viruses taken together also show an association with moderate differentiation.

#### Loss of heterozygosity in 3p

Figure 2

**Figure 2:**
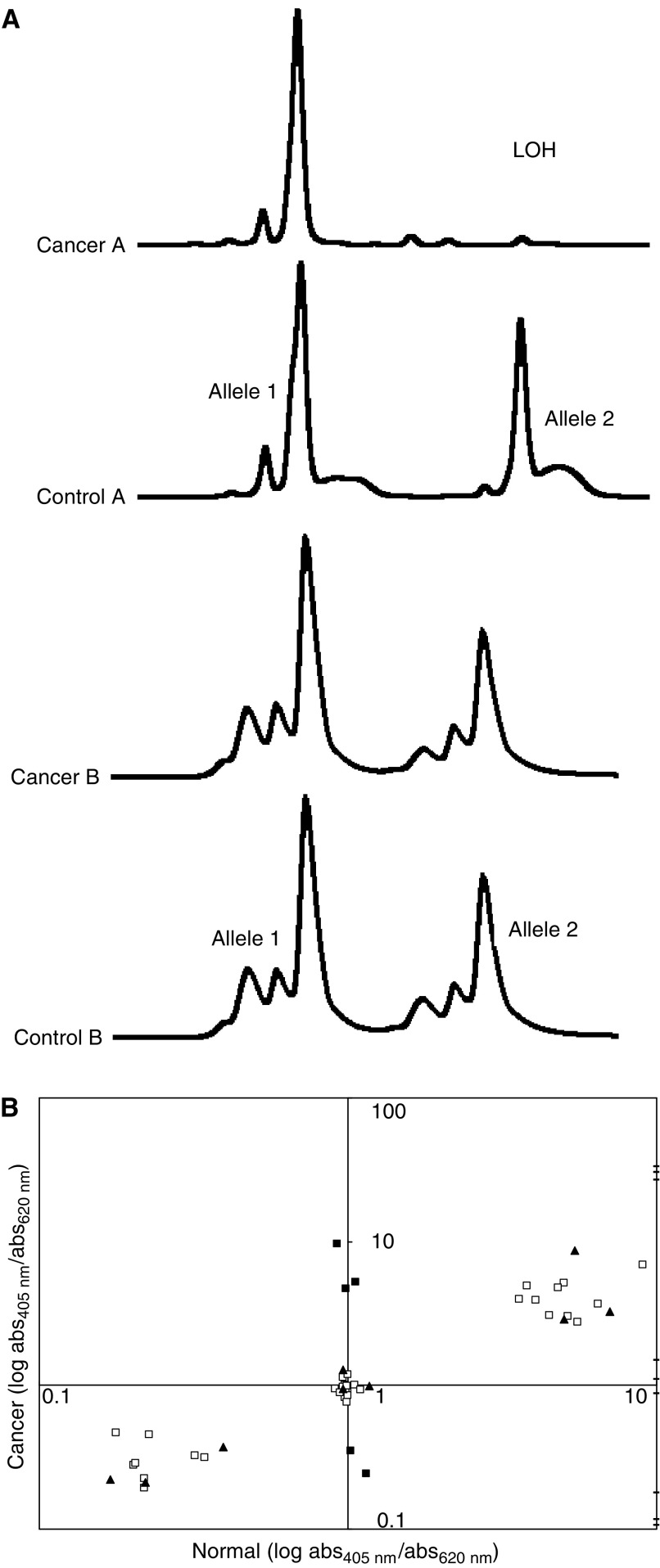
(**A**) Loss of heterozygosity in cancer A but not in cancer B at the D3S1300 locus as detected by microsatellite analysis. (**B**) Loss of heterozygosity at the rs722070 locus by SNP analysis. Results of a representative experiment involving 33 test samples. □, Homozygous cases or heterozygous cases showing no LOH. ▪, Five heterozygous cases showing LOH in the cancer, confirmed by repeat testing. **—**, Positive controls for allele 1, allele 2 and alleles 1+2 (supplied by the manufacturer) tested in triplicate. ▴, Nine control samples previously tested and confirmed to be homozygous or heterozygous at this locus.

shows representative LOH profiles obtained by microsatellite analysis (Figure 2A) and SNP analysis (Figure 2B). Loss of heterozygosity in 3p can occur over the entire length of the chromosome (Table 1) and the occurrence of LOH anywhere within 3p was associated with increasingly poor histological differentiation and with increased depth of invasion (Table 3a

**Table 3:** Significant associations between LOH in 3p and markers of cancer progression, HR HPV and FHIT protein expression in 83 cancers: (a) Allelic loss in any region of 3p; (b) allelic loss at specific 3p loci for cancers informative at that locus calculated

		**Total**		**Risk marker positive**		**Statistical analysis**		
**LOH site**	**Risk markers**	**No LOH**	**With LOH**	**No LOH**	**With LOH**	***P*-value**	**OR**	**95% CI**
*(a) For all cancers*								
Anywhere on 3p	Differentiation – well	36	47	6	1		1.00	
	– moderate	36	47	19	23		7.26	
	– poor	36	47	11	23		12.55	
						*P*-trend=0.018		
	Depth ⩾10 mm	36	47	14	40	2*P*⩽0.001	8.980	2.848–29.751
*(b) For informative cancers only*								
FHIT intron 3	Lympn node involvement	28	10	5	8	2*P*<0.001	18.400	2.382–206.044
FHIT intron 5 (SNP)	Depth ⩾10 mm	28	10	18	10	2*P*=0.038	a	1.305–a
FHIT intron 5 (MS)	Depth ⩾10 mm	54	20	31	20	2*P*<0.001	a	3.707–a
	Abnormal FHIT protein	54	20	25	16	2*P*=0.017	4.640	1.247–21.169

a Cannot be calculated.

). Overall, LOH was found in 57% cancers and the number of loci lost ranged from 1 to 11 (Figure 3

**Figure 3:**
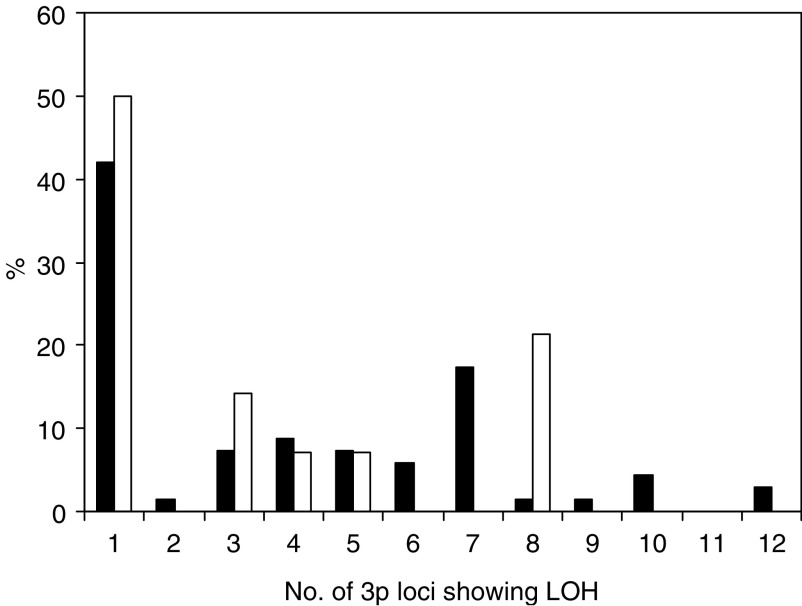
The number of 3p loci with LOH in individual cancers. ▪ Group A (including HPV16) □ HPV18.

). No significant association was found between LOH in the MLH1 mismatch repair gene and markers of cancer progression.

#### FHIT protein expression

Genomic instability in the FHIT gene was associated with loss of FHIT expression in a total of two loci out of seven examined, both located within in intron 5 (Table 3b). The loss of FHIT protein expression was not correlated with any of the markers of cancer progression studied.

### Evaluation of HR HPV, 3p LOH and FHIT expression in CIN1 and CIN1^*^ lesions

A total of 29 CIN1 lesions were positive for HR HPV, of which 10 contained HPV16. The incidences of HPV16, HR HPV as a group and LOH in and close to FHIT intron 5 were not significantly different in the two CIN1 categories, but the incidence of abnormal FHIT protein expression was significantly higher in CIN1^*^ (38 *vs* 10%) (Tables 4

**Table 4:** Cancer prognostic markers in CIN1 and CIN1^*^ lesions

	**CIN1 lesions**	CIN1* lesions	
**Parameters**	**(*n*=50)**	**(*n*=24)**	***2p***
HR HPV	5	5	0.277
16	4	2	1.000
18	10	3	0.528
Others^a^	31	14	0.803
None			
			
D3S1300 locus	5	4	0.216
LOH	35	10	
HET/no LOH			
			
D3S1582 locus	2	3	0.314
LOH	39	16	
HET/no LOH			
			
FHIT protein	45	15	0.009
Present			
Absent or reduced	5	9	

a Other types detected were HPV 6, 11, 18, 30, 31, 45, 52, 53, 56, 58, 66 and 67.

and 5

**Table 5:** Association between co-existing lesion grades and FHIT or PCNA protein status of CIN and CIN1^*^ lesions

		**CIN1 lesion status**					
		**FHIT**			**PCNA**		
**Co-existing lesion**	**Total**	**Abnormal**	***P*_trend_**	**OR**	**Positive**	***P*_trend_**	**OR**
None	50	5		1.00	35		1.00
							
CIN1/CIN2	3	1		4.50	2		0.86
							
CIN2	12	5		6.43	6		0.43
							
CIN3	9	3	0.010	4.50	8	0.861	3.43

).

## DISCUSSION

In all, 93% of cervical cancers contained HR HPV. HPV16 was the most common and was detected in 61% cancers overall and 71% of squamous carcinomas. Although HR HPV is known to be pivotal in the development of cervical cancer, its role in cancer progression as assessed here for HPV16 against histological markers was found to be limited to an association with vascular involvement. HPV16 occurred more frequently in moderately differentiated cancers and did not show any preference for adenosquamous carcinoma, a histological type which is usually poorly differentiated and has a lower 5-year survival rate (Davy *et al*, 2003; Farley *et al*, 2003). In contrast, LOH in 3p was found to be associated with poor differentiation, increased depth of invasion and, when the LOH occurred in FHIT intron 3 with lymph node involvement or in FHIT intron 5 with abnormal FHIT protein expression (Table 3b). Abnormal FHIT protein expression was not associated with any of the markers of cancer progression examined. This suggests that chromosomal instability alone may be an underlying factor in the histological manifestation of markers of cancer progression observed.

It is of current interest to find markers which can identify those CIN1 lesions which are likely to progress to more severe grades, and HR HPV and the FHIT gene are two likely candidates. In this study, we considered that CIN1^*^ lesions should indicate a greater potential for progression because there is good evidence to suggest that co-existing lesions, even when topographically distinct, could have a common origin and could be under the influence of the same ‘field’ effect (Larson *et al*, 1977; Park *et al*, 1998) and abnormal FHIT protein expression has been found to occur more frequently in CIN2/3 lesions co-existing with cancer (Connolly *et al*, 2000). Our results show that CIN1 and CIN1^*^ lesions have a similar incidence of HPV16 or other HR HPV (Table 4) so that the detection of HR HPV *per se* may not be sufficient to predict progression of CIN1. Interestingly, the incidence of abnormal FHIT protein expression in CIN1^*^ is similar to that reported for CIN2/3 lesions (Birrer *et al*, 1999; Connolly *et al*, 2000; Terry *et al*, 2002) and it was also correlated with the severity of the initially co-existing lesion grades (Table 5). If these findings are confirmed by a larger study, abnormal expression of FHIT protein in either biopsies (as in this study) or in cervical smears (Vecchione *et al*, 2001) could represent an additional marker for progressive potential in CIN1 lesions. Replacement therapy for the FHIT protein might also be effective in preventing the progression of CIN1 to higher grade lesions.

## References

[bib1] Acevedo CM, Henriquez M, Emmert-Buck MR, Chuaqui RF (2002) Loss of heterozygosity on chromosome arms 3p and 6q in microdissected adenocarcinomas of the uterine cervix and adenocarcinoma *in situ*. Cancer 94: 793–802 11857315 10.1002/cncr.10275

[bib2] Birrer MJ, Hendricks D, Farley J, Sundborg MJ, Bonome T, Walts MJ, Geradts J (1999) Abnormal Fhit expression in malignant and premalignant lesions of the cervix. Cancer Res 59: 5270–5274 10537308

[bib3] Borysiewicz LK, Fiander A, Nimako M, Man S, Wilkinson GW, Westmoreland D, Evans AS, Adams M, Stacey SN, Boursnell ME, Rutherford E, Hickling JK, Inglis SC (1996) A recombinant vaccinia virus encoding human papillomavirus types 16 and 18, E6 and E7 proteins as immunotherapy for cervical cancer. Lancet 347: 1523–1527 8684105 10.1016/s0140-6736(96)90674-1

[bib4] Chung TKH, Cheung TH, Lo WK, Yu MY, Hampton GM, Wong HKT, Wong YF (2000) Loss of heterozygosity at the short arm of chromosome 3 in microdissected cervical intraepithelial neoplasia. Cancer Lett 154: 189–194 10806307 10.1016/s0304-3835(00)00398-0

[bib5] Connolly DC, Greenspan DL, Wu R, Ren X, Dunn RL, Shah KV, Jones RW, Bosch FX, Munoz N, Cho KR (2000) The loss of Fhit expression in invasive cervical carcinomas and intraepithelial lesions associated with invasive disease. Clin Cancer Res 6: 3505–3510 10999736

[bib6] Corbin S, Neilly ME, Espinosa III R, Davis EM, McKeithan TW, Le Beau MM (2002) Identification of unstable sequences within the common fragile site at 3p14.2: implications for the mechanism of deletions within fragile histidine triad gene/common fragile site at 3p14.2 in tumors. Cancer Res 62: 3477–3484 12067991

[bib7] Cuzick J, Terry G, Ho L, Monaghan J, Lopes A, Clarkson P, Ducan I (2000) Association between high-risk HPV types, HLA DRB1^*^ and DQB1^*^ alleles and cervical cancer in British women. Br J Cancer 82: 1348–1352 10755413 10.1054/bjoc.1999.1103 PMC2374489

[bib8] Davy MLJ, Dodd TJ, Luke CG, Roder DM (2003) Cervical cancer: effect of glandular cell type on prognosis, treatment, and survival. Obstet Gynecol 101: 38–45 12517643 10.1016/s0029-7844(02)02275-5

[bib9] Druck T, Kastury K, Hadaczek P, Podolski J, Toloczko A, Sikorski A, Ohta M, LaForgia S, Lasota J, McCue P (1995) Loss of heterozygosity at the familial RCC t(3;8) locus in most clear cell renal carcinomas. Cancer Res 55: 5348–5353 7585599

[bib10] Dumon KR, Ishii H, Vecchione A, Trapasso F, Baldassarre G, Chakrani F, Druck T, Rosato EF, Williams NN, Baffa R, During MJ, Huebner K, Croce CM (2001) Fragile histidine triad expression delays tumor development and induces apoptosis in human pancreatic cancer. Cancer Res 61: 4827–4836 11406559

[bib11] Farley JH, Hickey KW, Carlson JW, Rose GS, Kost ER, Harrison TA (2003) Adenosquamous histology predicts a poor outcome for patients with advanced-stage, but not early-stage, cervical carcinoma. Cancer 97: 2196–2202 12712471 10.1002/cncr.11371

[bib12] Glover TW, Stein CK (1988) Chromosome breakage and recombination at fragile sites. Am J Hum Genet 43: 265–273 3137811 PMC1715373

[bib13] Guo Z, Wu F, Asplund A, Hu X, Mazurenko N, Kisseljov F, Pontén J, Wilander E (2001) Analysis of intratumoral heterogeneity of chromosome 3p deletions and genetic evidence of polyclonal origin of cervical squamous carcinoma. Mod Pathol 14: 54–61 11235906 10.1038/modpathol.3880256

[bib14] Hibi K, Takahashi T, Yamakawa K, Ueda R, Sekido Y, Ariyoshi Y, Suyama M, Takagi H, Nakamura Y, Takahashi T (1992) Three distinct regions involved in 3p deletions in human lung cancer. Oncogene 7: 445–449 1347916

[bib15] Ji L, Fang B, Yen N, Fong K, Minna JD, Roth JA (1999) Induction of apoptosis and inhibition of tumorigenicity and tumor growth by adenovirus vector-mediated fragile histidine triad (FHIT) gene overexpression. Cancer Res 59: 3333–3339 10416589

[bib16] Kastury K, Baffa R, Druck T, Ohta M, Cotticelli MG, Inoue H, Negrini M, Rugge M, Huang D, Croce CM, Palazzo J, Huebner K (1996) Potential gastrointestinal tumor suppressor locus at the 3p14.2 FRA3B site identified by homozygous deletions in tumor cell lines. Cancer Res 56: 978–983 8640789

[bib17] Kleter B, Doorn LJ, Schegget J, Schrauwen L, Krimpen K, Burger M, Harmsel B, Quint W (1998) Novel short-fragment PCR assay for highly sensitive broad-spectrum detection of anogenital human papillomaviruses. Am J Pathol 153: 1731–1739 9846964 10.1016/S0002-9440(10)65688-X PMC1866345

[bib18] Larson AA, Liao SY, Stanbridge EJ, Cavenee WK, Hampton GM (1977) Genetic alterations accumulate during cervical tumorigenesis and indicate a common origin for multifocal lesions. Cancer Res 57: 4171–4176 9331069

[bib19] Li J, Yen C, Liaw D, Podsypanina K, Bose S, Wang SI, Puc J, Miliaresis C, Rodgers L, McCombie R, Bigner SH, Giovanella BC, Ittmann M, Tycko B, Hibshoosh H, Wigler MH, Parsons R (1997) PTEN, a putative protein tyrosine phosphatase gene mutated in human brain, breast, and prostate cancer. Science 275: 1943–1947 9072974 10.1126/science.275.5308.1943

[bib20] Munoz N, Bosch FX, de Sanjose S, Herrero R, Castellsague X, Shah KV, Snijders PJ, Meijer CJ, International Agency for Research on Cancer Multicenter Cervical Cancer Study Group (2003) Epidemiologic classification of human papillomavirus types associated with cervical cancer. N Engl J Med 348: 518–527 12571259 10.1056/NEJMoa021641

[bib21] Naylor SL, Johnson BE, Minna JD, Sakaguchi AY (1987) Loss of heterozygosity of chromosome 3p markers in small cell lung cancer. Nature 329: 451–454 2821400 10.1038/329451a0

[bib22] Park J, Sun D, Genest DR, Trivijitsilp P, Suh I, Crum CP (1998) Coexistence of low and high grade squamous intraepithelial lesions of the cervix: morphologic progression or multiple papillomaviruses? J Gynecol Oncol 70: 386–391 10.1006/gyno.1998.5100 9790792

[bib23] Roz L, Gramegna M, Ishii H, Croce CM, Sozzi G (2002) Restoration of fragile histidine thriad (FHIT) expression induces apoptosis and suppresses tumorigenicity in lung and cervical cancer cell lines. Proc Natl Acad Sci USA 99: 3615–3620 11891319 10.1073/pnas.062030799 PMC122572

[bib24] Sard L, Accomero P, Tornielli S, Delia D, Bunone G, Campiglio M, Colombo MP, Gramegna M, Croce CM, Pierotti MA, Sozzi G (1999) The tumor-suppressor gene FHIT is involved in the regulation of apoptosis and in cell cycle control. Proc Natl Acad Sci USA 96: 8489–8492 10411902 10.1073/pnas.96.15.8489 PMC17543

[bib25] Schantz SP, Huang Q, Shah K, Murty VVVS, Hsu TC, Yu Guopei G, Andersen PE, Huvos AG, Chaganti RSK (2000) Mutagen sensitivity and environmental exposures as contributing causes of chromosome 3p losses in head and neck cancers. Carcinogenesis 21: 1239–1246 10837016

[bib26] Terry G, Ho L, Londesborough P, Cuzick J (2002) Abnormal FHIT expression profiles in cervical intraepithelial neoplastic (CIN) lesions. Br J Cancer 86: 376–381 11875703 10.1038/sj.bjc.6600077 PMC2375220

[bib27] Thorland EC, Myers SL, Persing DH, Sarkar G, McGovern RM, Gostout BS, Smith DI (2000) Human papillomavirus type 16 integrations in cervical tumors frequently occur in common fragile sites. Cancer Res 60: 5916–5921 11085503

[bib28] Vecchione A, Zanesi N, Trombetta G, French D, Visca P, Pisani T, Botti C, Vecchione A, Croce CM, Mancini R (2001) Cervical dysplasia, ploidy and human papillomavirus status correlate with loss of FHIT expression. Clin Cancer Res 7: 1306–1312 11350899

[bib29] Wistuba II, Montellano FD, Milchgrub S, Virmani AK, Behrens C, Chen H, Ahmadian M, Nowak JA, Muller C, Minna JD, Gazdar AF (1997) Deletions of chromosome 3p are frequent and early events in the pathogenesis of uterine cervical carcinoma. Cancer Res 57: 3154–3158 9242443

[bib30] Ylitalo N, Josefsson A, Melbye M, Sorensen P, Frisch M, Andersen PK, Sparen P, Gustafsson M, Magnusson P, Ponten J, Gyllensten U, Adami H (2000) A prospective study showing long-term infection with human papillomavirus 16 before the development of cervical carcinoma *in situ*. Cancer Res 60: 6027–6032 11085523

